# Fabrication of elastic, conductive, wear-resistant superhydrophobic composite material

**DOI:** 10.1038/s41598-021-92231-x

**Published:** 2021-06-16

**Authors:** Seyed Mehran Mirmohammadi, Sasha Hoshian, Ville P. Jokinen, Sami Franssila

**Affiliations:** 1 Department of Chemistry and Materials Science, Micronova Nanofabrication Centre, Aalto University, 02150 Espoo, Finland; 2 Advacam Ltd, Micronova Nanofabrication Centre, 02150 Espoo, Finland

**Keywords:** Polymers, Wetting, Mechanical properties, Synthesis and processing, Design, synthesis and processing

## Abstract

A polydimethylsiloxane (PDMS)/Cu superhydrophobic composite material is fabricated by wet etching, electroless plating, and polymer casting. The surface topography of the material emerges from hierarchical micro/nanoscale structures of etched aluminum, which are rigorously copied by plated copper. The resulting material is superhydrophobic (contact angle > 170°, sliding angle < 7° with 7 µL droplets), electrically conductive, elastic and wear resistant. The mechanical durability of both the superhydrophobicity and the metallic conductivity are the key advantages of this material. The material is robust against mechanical abrasion (1000 cycles): the contact angles were only marginally lowered, the sliding angles remained below 10°, and the material retained its superhydrophobicity. The resistivity varied from 0.7 × 10^–5^ Ωm (virgin) to 5 × 10^–5^ Ωm (1000 abrasion cycles) and 30 × 10^–5^ Ωm (3000 abrasion cycles). The material also underwent 10,000 cycles of stretching and bending, which led to only minor changes in superhydrophobicity and the resistivity remained below 90 × 10^–5^ Ωm.

## Introduction

Superhydrophobic surfaces (SHS) have high contact angles (CAs > 150°), low sliding angles (SAs < 10°), and low contact angle hysteresis (CAH)^1–3^. The lure of SHS owes to their potential applications in a wide range of fields, including desalination and anti-biofouling^2^, energy devices^3^, biomedical devices^4^, and heat transfer^5^.

Two different models describe the wetting behavior of liquid droplets on a structured surface: Wenzel (penetration) and Cassie-Baxter (CB) (suspension) states. In Wenzel state, both hysteresis and sliding angles are high due to the penetration of the droplet into the pores of the surface structure. In the Cassie-Baxter state, trapped air within the micro/nanostructures, called the plastron, decreases the contact area between a droplet and the solid surface. Consequently, the droplet can easily roll off the surface at very small tilting angles^1–3^.

The main factors of superhydrophobicity are surface roughness (surface topography), surface chemistry, the surface tension of the test liquid, and the CB state^5–7^. SHS can be fabricated via two main approaches: (1) adding low surface energy coating, such as fluoropolymers, to a structured surface, and (2) creating hierarchical micro/nanoscale topography of hydrophobic materials. The former method has been extensively applied to metal topographies such as etched aluminum, anodic alumina, and boehmite nanostructures^8–14^. When coated by hydrophobic films, excellent superhydrophobicity and in some cases also oleophobicity has been shown. These surfaces display anticorrosion^8^ and resistance to alkaline and saline immersion, UV irradiation, and icing^13^. Fuoroalkylsilane (FDTS)-coated etched aluminum survived 50 tape peel tests but was not very good in alkaline and acidic tests^12^.

Hierarchical hydrophobic materials approach can lead to more robust superhydrophobic surfaces because the wear of the coating is not involved^15^. One powerful method for fabricating topographical surfaces of hydrophobic materials is polymer replication. The original structures are either natural objects like plant leaves^16,17^ or rough metal surfaces created by various etching and deposition techniques and their combinations^18–22^. Replicated materials include PDMS, polyurethane (PU), high-density polyethylene (HDPE), epoxies, ultra-high-molecular-weight polyethylene (UHMWPE), Polytetrafluoroethylene (PTFE), and CYTOP. Even though the polymer replica surfaces display high contact angles, they are often in Wenzel state with large sliding angles^20^. Therefore, fluoropolymer coatings are often employed^21,23^, and again the problem of film durability is encountered.

A few superhydrophobic replicas without coatings have been demonstrated. HDPE replicated from etched aluminum showed excellent chemical durability and self-cleaning effect^19^. PDMS, PU, UHMWPE, and PTFE surfaces replicated from aluminum and alumina masters exhibited superhydrophobicity, but these films were not subjected to environmental or mechanical tests^18^. PDMS replicas displayed > 150° contact angles and underwent 10 000 pushing and bending tests successfully; however, sliding angles were > 90° for small droplets (5–10 µL), and only 70 µL droplets showed < 10° sliding angles^22^.

The limited mechanical durability of superhydrophobic surfaces is often their Achilles’ heel due to the presence of vulnerable micro/nanostructures. Elastic materials are preferred over plastic and brittle materials because they survive abrasive tests better^20^. Epoxy pillars were covered by carbon nanotubes (CNT), which survived mechanical loads due to CNT elasticity^24^.

As micro/nanostructures experience damage, the contact angle hysteresis increases, resulting in an increase of the sliding angle values^15,25^. Zhu et al*.* showed that when PDMS-coated copper mesh was subjected to 100 cycles of abrasion with the silicon carbide paper and load of 1 kg, the contact angle decreased from 156° to 139° due to the destruction of nanostructures^26^. Long et al*.* reported that the water contact angle of a PDMS-coated hierarchical aluminum surface decreased from 158° to 125° and the sliding angle rose from 2° to 90° after the abrasion experiments^27^. Other polymer-coated superhydrophobic surfaces have been subjected to abrasive tests, but under very mild conditions: 4 N load^13^, and 100 g load^10^. Additional metrics for durability have been proposed by Malavasi et al., including resistance to heating, alcohol immersion, and hydrocarbon immersion^11^.

Electrical conductivity combined with superhydrophobicity has been demonstrated before, but not with polymer replication techniques. Several of the previous works have utilized carbon nanotube-based conductive superhydrophobic surfaces^28,29^. Zou et al*.* reported a thin (1 μm) layer of CNT and conductive polymer that were mixed to form a conductive layer for volatile organic vapor sensing with minimized moisture interference^28^. Yao et al. showed conductive superhydrophobic nanocomposite coatings by spraying SiO_2_ nanoparticles and fluorinated multiwalled carbon nanotubes (MWCNTs) on a polymer substrate^29^. However, the mechanical durability of superhydrophobicity or conductivity was not reported in either of these works. Other routes to conductive superhydrophobic surfaces have included a porous electrodeposited zinc oxide film coated with a fluorinated monolayer^30^, and silver nanowires/particles also coated with a fluorinated monolayer^31^. However, due to the use of the monolayer hydrophobic coating, the mechanical durability is likely very limited.

Previously, we have shown superhydrophobic TiO_2_/PDMS replicas from aluminum that were robust against mechanical abrasion and other environmental stressors^25^. They were replicated from etched aluminum which was coated by atomic layer deposition (ALD) TiO_2_, and the TiO_2_ coating was transferred to PDMS when the aluminum substrate was sacrificially etched away. No fluoropolymer coating was used. The fabrication process is, however, limited by the requirement of ALD film, which is a costly step that is not available everywhere. Now, we show how a similar transfer process can create superhydrophobic surfaces that are not only robust against abrasion, stretch, and bending but also highly conductive.

We introduce copper electroless plating (ELP) using copper (II) sulfate pentahydrate (CuSO_4_.5H_2_O) in an acidic bath (pH ≈ 2–3), using sulfuric acid and hydrochloric acid. Previous ELP processes have been carried out under alkaline conditions^32,33^. Electroless plating is a simple, efficient, and low-cost method for depositing a metallic layer. It is based on a chemical reaction between a reducing agent and metal ions. The thickness of plated metal can be easily controlled by changing, e.g., plating time and/or temperature^34–36^. Many types of bath solutions and reducing agents have been employed for copper electroless plating, including glyoxylic acid (pH ≈ 12)^32^, formaldehyde alkaline solution bath (pH ≈ 12)^33^, dimethylamine borane (pH ≈ 7.5)^37^. It has been found that low pH (< 7) baths result in uniform and smooth Cu film with adequate adhesion and low resistivity^37,38^. Hypophosphite-based bath is another solution (pH 9); however, it needs nickel ions for Cu plating^38^.

Our process is composed of three main steps: aluminum etching, copper electroless plating, and PDMS casting on top of the plated copper. The aluminum substrate is then completely etched away, resulting in a PDMS-backed copper surface with topography defined by etched aluminum micro and nanostructures. The resulting material exhibits multiple size scales from tens of nanometers to tens of micrometers and can tolerate stretching (up to 10,000 cycles), abrasion (up to 1500 cycles), and bending (up to 10,000 cycles) with negligible effect on superhydrophobicity and only slightly reduced conductivity. No fluorine compounds are used, and the elimination of thin coatings is essential for durability.

## Results and discussion

### Fabrication of PDMS/Cu composite material

**Figure 1:**
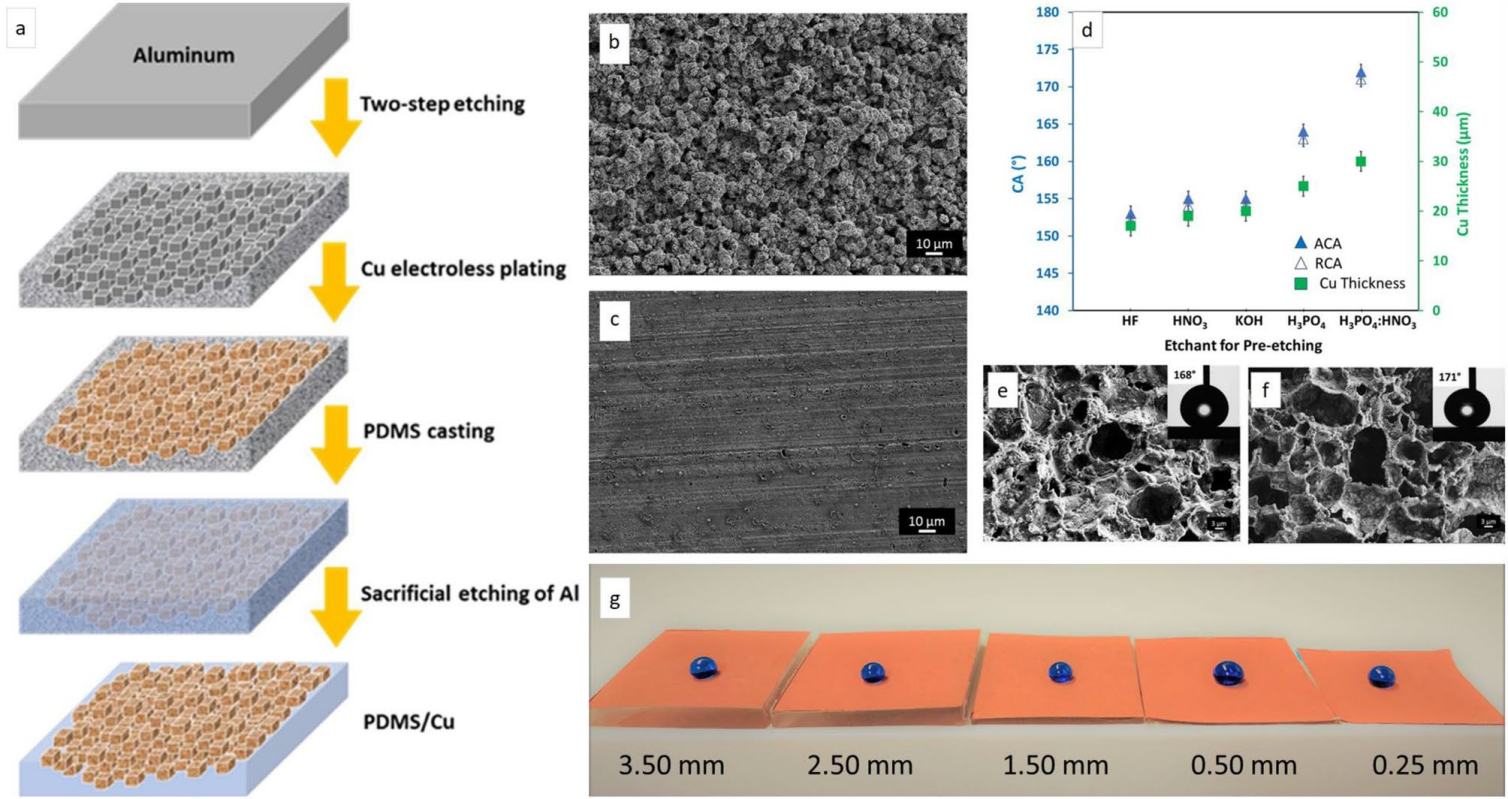
(**a**) Schematic illustration of the fabrication process to obtain elastic, conductive, wear-resistant superhydrophobic composite PDMS/Cu material. The SEM images of 12 min single-step HCl etched Al at two different HCl concentrations: in (**b**) 3 M HCl, microscale protrusions were obtained and (**c**) 1 M HCl showing mostly unaffected surface. (**d**) The effect of etchant types used as a pre-etching step on contact angles and plated copper thickness (1 h plating at 20 °C). The SEM images show the morphologies of the PDMS/Cu surface obtained by (**e**) one-step and (**f**) two-step etching of Al substrate and one-hour copper plating. The two-step etched sample shows richer detail of micro and nanostructures. Corresponding advancing contact angles are shown in insets. (**g**) Digital photograph of composite with different thicknesses backing layer 3.50–0.25 mm displaying water contact angle around 170°.

Figure 1a shows the schematic of our fabrication process. The substrate was a 0.4 mm thick aluminum plate (Al 6061-T6, 12.0" × 12.0", Online Metals). The plate was cleaned by acetone and isopropanol, followed by etching in a two-step process. First, Al was etched for three minutes in a phosphoric acid-based solution to remove surface oxide. After rinsing the sample with deionized (DI) water, the second etching was done in HCl for nine minutes to obtain hierarchical micro and nanostructures. Then, electroless plating was conducted in the acidic copper bath at room temperature. Table 1 summarizes the etching and plating conditions. The chemicals were purchased from Sigma Aldrich. After copper plating, PDMS (Sylgard 184 Silicone Elastomer Kit, Dow Chemical Co.) casting with the ratio of 10:1 (monomer to the crosslinking agent) was done on top of the plated copper film. The sample was cured at 65 °C for 3 h in an oven. Finally, the aluminum was removed by sacrificial etching in 6 M HCl for 10 min, followed by rinsing with DI water, drying at room temperature, and final curing at 65 °C for 2 h in an oven.

**Table 1:** The conditions of pre-etching, HCl etching, and the copper electroless plating bath.

Process	Chemicals	Volume ratios and concentrations	Amount	Time (min)
Pre-etching	H_3_PO_4_:HNO_3_:H_2_O	20:1:5	–	3
Etching	HCl	3 M	–	9
	CuSO_4_·5H_2_O	98%	18 g	
	H_2_SO_4_	95–97%	28.8 mL	
Copper	HCl	37%	17.5 µL	60
ELP	PEG^a^	–	90 mg	
	SPS^b^	–	1 mg	
	DI water	–	270 mL	

^a^Polyethylene glycol.

^b^Bis-(sodium sulfopropyl)-disulfide.

### Aluminum etching process

The first step was to optimize the aluminum wet etching conditions for enabling maximal superhydrophobicity of the final PDMS/Cu composite. One-step and two-step aluminum etching processes were explored to show the effect of the etching process on surface morphologies and contact angle values. The morphology and contact angles were evaluated from the final PDMS/Cu composite material.

In the one-step etching process, an aluminum substrate was etched in 1 M, 3 M, and 6 M HCl solutions for 12 min. It was found that a rough structure with microscale protrusions can be obtained by using 3 M HCl solution in the one-step etching process (Fig. 1b), while no significant roughness was achieved in 1 M HCl solution (Fig. 1c). Aluminum was completely detached by 6 M HCl in 12 min.

In the two-step etching technique, a pre-etching step was used for three minutes before HCl etching. We tested different types of etchants to investigate an appropriate type of solution for the pre-etching step, as provided in Table 2. The results show the mixture of phosphoric acid, nitric acid, and DI water (a typical Al wet etchant used in microfabrication) resulted in the highest contact angle values of the final material, around 170° for both advancing and receding contact angles (ACA and RCA) (Fig. 1d). The depth and size of structures can be controlled by changing etching conditions, such as the etchant type, concentration of etchant, time, and temperature of the etching process^39–41^. Similar results were obtained by Fernandez et al*.* who showed that a combination of different concentrations of HCl in the two-step etching process led to the uniform textured surfaces^40^. This can be explained by increasing the height/depth of structures obtained from the two-step etching process.

**Table 2:** The etchants used for the pre-etching step and their volume ratios and etching time.

Etchants for pre-etching	Volume ratios	Time (min)
HF:H_2_O	1:3	3
HNO_3_:H_2_O	1:3	3
KOH:H_2_O	1:3	3
H_3_PO_4_:HNO_3_:H_2_O	20:1:5	3

### Copper electroless plating

Copper electroless plating was done on etched Al samples. Figure 1d shows the effect of the pre-etching step on the contact angle and plated copper thickness after 1 h of plating at room temperature. The contact angles were measured after a complete process that involved aluminum etching, Cu plating, PDMS curing, and sacrificial etching of aluminum.

Copper plating on 2-step etched Al resulted in 30 ± 2 μm thick film after 1 h, whereas the thickness was 20 ± 2 μm for the one-step etched sample. The thicker copper film shows more pronounced nanoscale surface features and resulted in improvement of the CA values, as can be seen in Fig. 1e,f. Therefore, we chose to utilize the phosphoric-nitric acid pre-etching in all following experiments.

**Figure 2:**
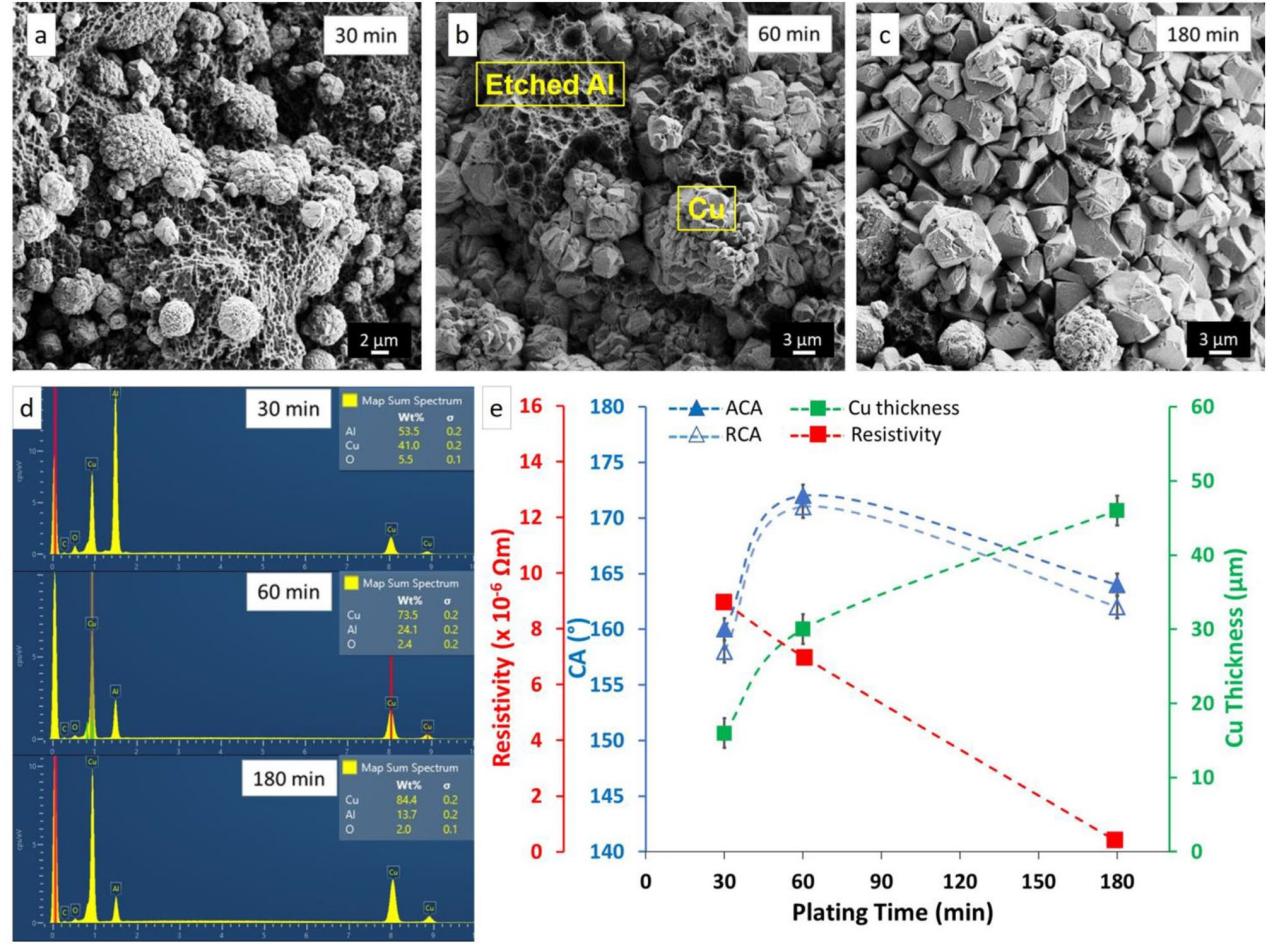
Morphologies of Cu on etched Al after (**a**) 30 min, (**b**) 60 min, and (**c**) 180 min of electroless plating. The morphology of the plated Cu changed from micro-particles to a continuous film with increasing plating time. (**d**) EDX analysis of Al/Cu samples after 30 min, 60 min, and 180 min of Cu electroless plating. Even after 180 min of Cu plating, some Al is detectable, indicating that the copper film coverage is not full 100%. (**e**) The effect of copper plating time on CAs, copper thickness, and resistivity of the resulting PDMS/Cu material.

To investigate the effect of Cu plating time on the morphology, CA values, and conductivity of PDMS/Cu material, samples were prepared with different plating times: 30, 60, and 180 min (Fig. 2a–c). The morphology of the plated copper changed from micro-particles to a continuous film and the thickness of plated copper increased from 16 to 46 μm as plating time increased from 30 to 180 min. After 30 min of plating, copper micro-particles are clearly distinguishable on the etched aluminum surface (Fig. 2a). After 60 min of plating, although copper formed a mostly continuous film, some aluminum is still visible (Fig. 2b). These non-coated areas will allow PDMS on the surface after sacrificial etching of aluminum, resulting in composite PDMS/Cu surface. Increasing plating time to 180 min leads to formation of almost fully continuous copper layer, in which copper particles have coalesced and grown on top of each other (Fig. 2c). PDMS is not able to diffuse through the thicker copper film, resulting in a decrease of contact angle values.

This scanning electron microscopy (SEM) analysis is supported by energy-dispersive X-ray spectroscopy (EDX) (Fig. 2d); the content of Al decreased from 53.5 to 13.7 wt% when the plating time increased from 30 to 180 min. The content of Cu, on the other hand, increased from 41.0 to 84.4 wt%, which indicates that Al was almost covered by a continuous Cu layer. Samples of Al/Cu without PDMS were used for EDX analyses.

Copper plating time and the corresponding copper thicknesses, CAs, and the resistivity of the final material can be seen in Fig. 2e. Increasing the copper thickness from 16 to 30 μm resulted in the increase of contact angles and reduction of resistivity. The plating time of 30 min resulted in a discontinuous copper film with a rather high resistivity. Increasing the thickness of copper from 16 to 30 μm resulted in a reduction of resistivity to 0.7 × 10^–5^ Ωm which is metallic conductivity, even though it is roughly 400 times higher than that resistivity of bulk copper. Based on the results in Fig. 2, we chose to focus on the material with 60 min Cu deposition to maximize the superhydrophobicity. Some other applications might benefit from longer deposition times to achieve lower resistivity at the expense of somewhat lower superhydrophobicity. Deposition times of less than 60 min are clearly suboptimal for both parameters. Su et al*.* obtained resistivity of ca. 30 × 10^–5^ Ωm for their 84 μm thick Ag-nanoparticle composite^42^ (40 × higher than ours, for a thicker layer), and sheet resistance of CNT-embedded SHS was 3 × 10^4^ Ω/sq^29^, while our samples display sheet resistances of 0.4 Ω/sq. Luo et al*.* reported resistivity of 1 × 10^–4^ Ωm^43^, and Wang et al*.* a resistivity of 2 Ωm for their superhydrophobic composite materials^44^, which are roughly 15 × and 100,000 × higher than our material, respectively. A superhydrophobic and conductive nanocomposite made of fluoropolymer and various allotropes of carbon exhibited resistivity of 1 × 10^–3^ Ωm^45^, but it was not subjected to any mechanical or environmental tests except water droplet impalement.

### Composite thickness tests

We also tested the actual thickness of the complete composite material (Fig. 1g). The superhydrophobic properties remain the same for all samples with different PDMS backing plate thicknesses, ranging from 0.25 to 3.50 mm. The copper thickness was 30 µm for all the samples. The water contact angles were in the range of 167°–172° advancing and 166°-171° receding for all samples indicating no detectable difference between the samples. We chose to perform rest of the experiments with PDMS thickness of 1.5 mm.

### Mechanical durability tests

The samples for mechanical durability tests were obtained by the two-step etching process and one-hour Cu electroless plating. We studied surface morphology, resistivity, and CAs of samples in a static stretch, cyclic stretch, abrasion cycle, and bending cycle tests.

#### Static stretch tests

**Figure 3:**
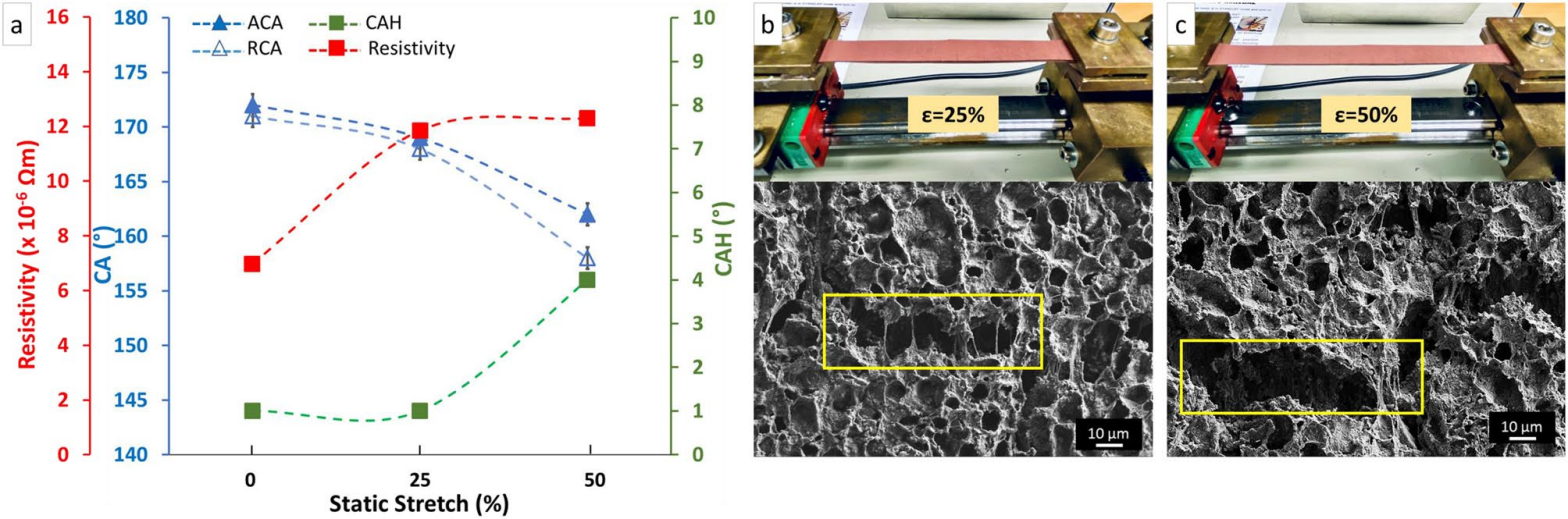
(**a**) The contact angles, contact angle hysteresis, and resistivity of non-stretched and stretched samples at 25% and 50% of tensile strain in a static stretch test. Photos of stretching test setup and the SEM images of static stretched samples at (**b**) 25% and (**c**) 50% of tensile values.

The contact angles, contact angle hysteresis, and resistivity values of non-stretched and stretched samples at 25% and 50% of tensile strain (ε) are plotted in Fig. 3a. These results are the CAs and resistivity of the material in a static stretched state.

The results indicate a slight change in the CAs of the PDMS/Cu material after 25% stretching; the advancing contact angle decreased from 172 ± 1° to 196 ± 1° and the receding contact angle decreased from 171 ± 1° to 168 ± 1°. For the stretched sample at 50% of tensile strain, the advancing contact angle decreased to 162 ± 1° and the receding contact angle to 158 ± 1°, resulting in an increase of contact angle hysteresis from 1° to 4°. The resistivity values increased from 0.7 × 10^–5^ to 1.2 × 10^–5^ Ωm for both 25% and 50% stretched samples.

The surface morphology of tensile stretched samples can be seen in Fig. 3b,c. The surface structures were preserved at 25% tensile strain but experienced deformation and distortion at 50% tensile strain. Contact angle hysteresis increase at 50% tensile strain is caused by damage to the hierarchical structures, as seen in Fig. 3c.

#### Cyclic stretch tests

**Figure 4:**
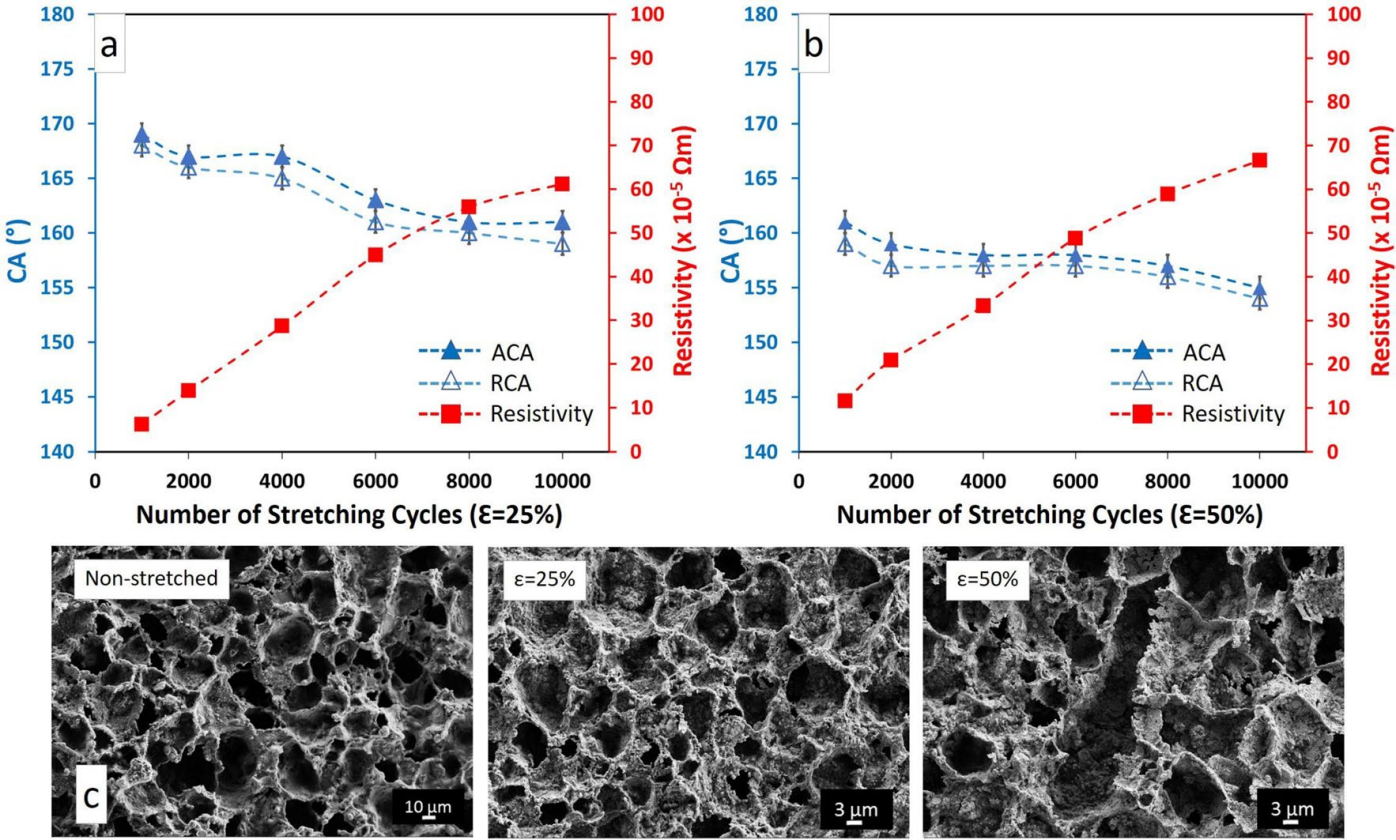
The contact angles and resistivity after being subjected to 10,000 stretching-relaxing cycles at (**a**) 25% and (**b**) 50% of tensile strain. (**c**) The SEM images of non-stretched and stretched samples after 10,000 stretching-relaxing cycles at 25% and 50% of tensile strain.

The advancing and receding contact angles and resistivity after cyclic stretch test of 1000 to 10,000 cycles are shown in Fig. 4a,b (Movie S1 in the Supplementary Material). After 10,000 cycles of stretching (25%), the advancing contact angle decreased from 172 ± 1° to 161 ± 1° and receding contact angle was reduced from 171 ± 1° to 159 ± 1° (Fig. 4a). In the case of 50% tensile strain, both advancing and receding contact angles declined to around 155 ± 1°, but superhydrophobic properties remained (Fig. 4b). The SA with 7 μL droplet was around 7° after 10,000 stretching-relaxing cycles at 25% tensile strain, same as the non-stretched sample. For 50% tensile strain, the sliding angle was 9°.

After 1000 stretching cycles, the resistivity values increased to 6 × 10^–5^ Ωm at 25% and to 12 × 10^–5^ Ωm at 50% of tensile strain. As the stretch cycles increased from 1000 to 10,000, the resistivity showed a clear upward trend and gradually increased to around 60 × 10^–5^ Ωm for both 25% and 50% of tensile strain, not dissimilar from 30 × 10^–5^ Ωm obtained by Su et al.^42^. A possible reason for this gradual increase can be explained by free monomers in PDMS. As the sample is stretched, these mobile monomers can increasingly penetrate into any cracks or boundaries opened up in the copper layer by stretching.

Besides, SEM images show that the surface morphologies of PDMS/Cu material after 10,000 cycles at ε = 25% and non-stretched sample are almost identical to each other. This means that hierarchical structures were preserved after 10,000 cycles of 25% stretching. There is slight deformation, but no change in overall topography can be observed after being subjected to the 10,000 stretching cycles at 50% tensile strain (Fig. 4c).

These results show that no serious damage is visible on the hierarchical structures of the surface after applying the cyclic stretch test and the superhydrophobicity of the PDMS/Cu material was preserved with CA > 150° and SA < 10°. In the case of silver-nanoparticle composite^42^, the contact angle remained above 150° and electrical resistance increased from 10 to 79 Ω after 200 cycles of stretching. If we estimate the resistivity increase of our samples at 200 stretch cycles from Fig. 4a,b, we end up with something close to the factor of ten, in accordance with silver-nanoparticle composite.

#### Abrasion tests

**Figure 5:**
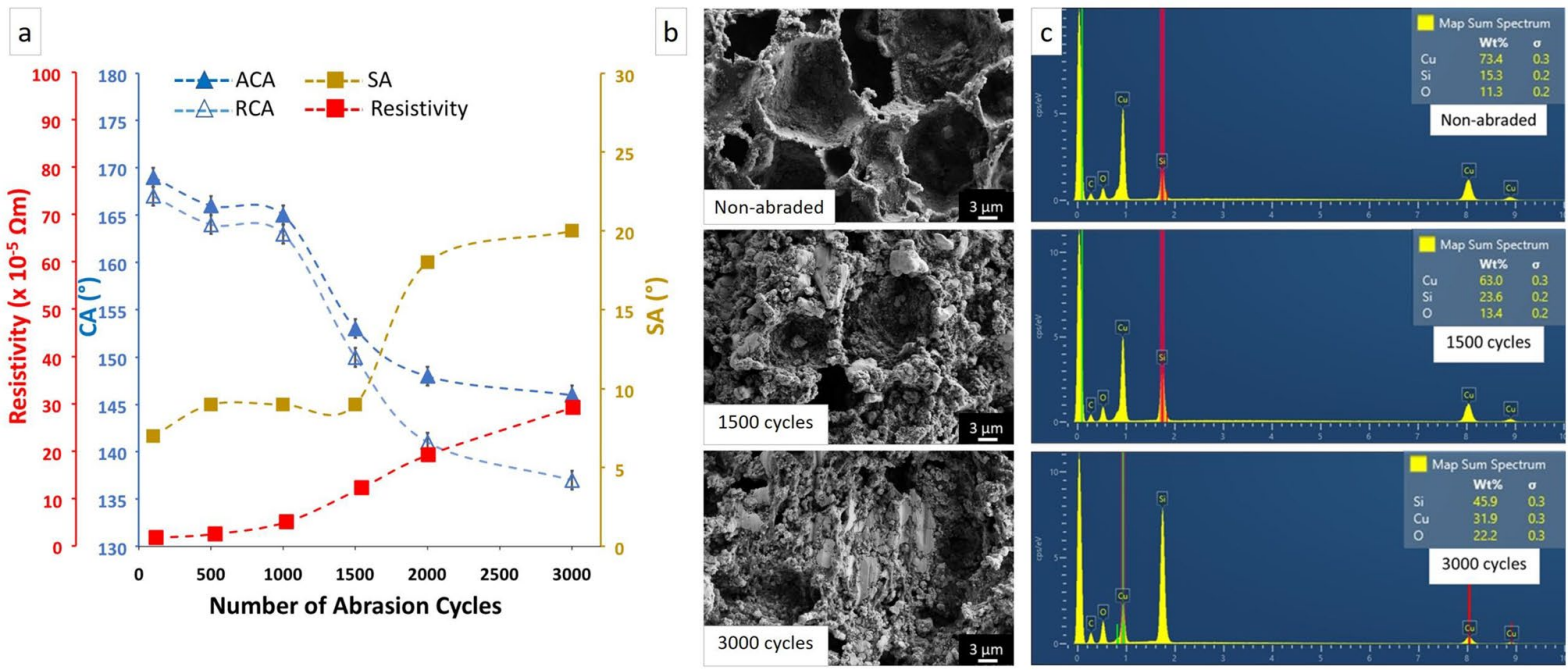
(**a**) The contact angles, sliding angles, and resistivity of abraded material from 100 to 3000 cycles. (**b**) The SEM images of surface structures of non-abraded and abraded materials after 1500 and 3000 cycles of abrasion. (**c**) EDX analysis of PDMS/Cu material for non-abraded, after 1500 and 3000 cycles of abrasion.

To investigate the wear resistance of the PDMS/Cu material abrasion cycle test was conducted using silicon carbide paper (P 1200) (Movie S2 in the supplementary material). The contact angles, sliding angles, and resistivity of the abraded surface at different cycles are given in Fig. 5a.

The advancing and receding contact angles decreased from around 170° to 150° after 1500 cycles, while no significant change in contact angle hysteresis and sliding angles was observed, and the material remained superhydrophobic. As the abrasion cycles increased beyond 1500 cycles, the contact angle hysteresis rose significantly from 2° to 9°, and SA increased from 7° to 20°.

Resistivity steadily increased from 0.7 × 10^–5^ Ωm (non-abraded) to 5 × 10^–5^ Ωm after 1000 cycles of abrasion. As the surface was subject to more abrasion cycles (> 1000), the resistivity increased to 30 × 10^–5^ Ωm after 3000 cycles due to the reduction of Cu content and the destruction of hierarchical structures. Wang et al. reported that when a fluorinated PDMS composite was subjected to the sandpaper-abrasion test, conductivity and superhydrophobicity were maintained for 80 abrasion cycles^44^, while our composite material maintains its properties for 1500 abrasion cycles. Wang et al. and Luo et al. showed that the static contact angle remained above 150° after 50–60 abrasion cycles^43,46^. However, no information was provided on the sliding angles or contact angle hysteresis, and thus the effect of abrasion on superhydrophobicity cannot be assessed.

The surface morphologies of non-abraded and abraded samples are shown in Fig. 5b. Although some rounding of structures is visible after 1500 cycles, the main surface features were preserved after 1500 cycles of sandpaper-abrasion test. As the material was subjected to 3000 cycles, destruction of finer structures can be observed and the superhydrophobicity was gradually lost.

The EDX analysis shows that the Cu content decreased slightly from 73.4 (non-abraded) to 63.0 wt% after 1500 cycles of abrasion and then significantly decreased to 31.9 wt% after 3000 cycles of abrasion due to the damage of hierarchical structures (Fig. 5c). The EDX results also show the increase in silicon and oxygen signals, which are likely due to more PDMS being visible after some of the copper was abraded away. On the other hand, the constant supply of PDMS monomers to the surface to coat freshly exposed copper is supposedly responsible for the long-lasting superhydrophobicity. This is very different compared to superhydrophobicity achieved via polymer coating: first of all, our polymer is mostly under the surface; and second, we have millimeter thickness, which is an ample supply. Nanostructured copper surface with copper oxide (CuO) has been found to exhibit low friction and improved wear resistance because wear debris is not on the surface but inside the microstructures^47^.

Only few conductive superhydrophobic surfaces have undergone mechanical durability tests. These include a fluorinated PDMS composite^44^ and a rubber composite^46^. The superhydrophobicity and conductivity were maintained after 1000 stretching cycles and 80 abrasion cycles for the PDMS composite^44^ and after 100 stretching cycles and 60 abrasion cycles for the rubber composite^46^. Luo et al*.* showed the static contact angle of polypropylene/polydopamine/Ag-nanoparticle/PDMS composite remained above 150° and conductivity decreased from 89.1 to 60.0 S/cm after 50 abrasion cycles (3X less conductive than our sample after 1000 abrasion cycles)^43^. Polymer-modified Ag-nanoparticles on natural rubber were reported to exhibit superhydrophobicity, elasticity, and conductivity without fluorination^42^. These structures were not subjected to abrasive tests, even though they survived kneading, torsion, and water impact tests.

#### Bending tests

**Figure 6:**
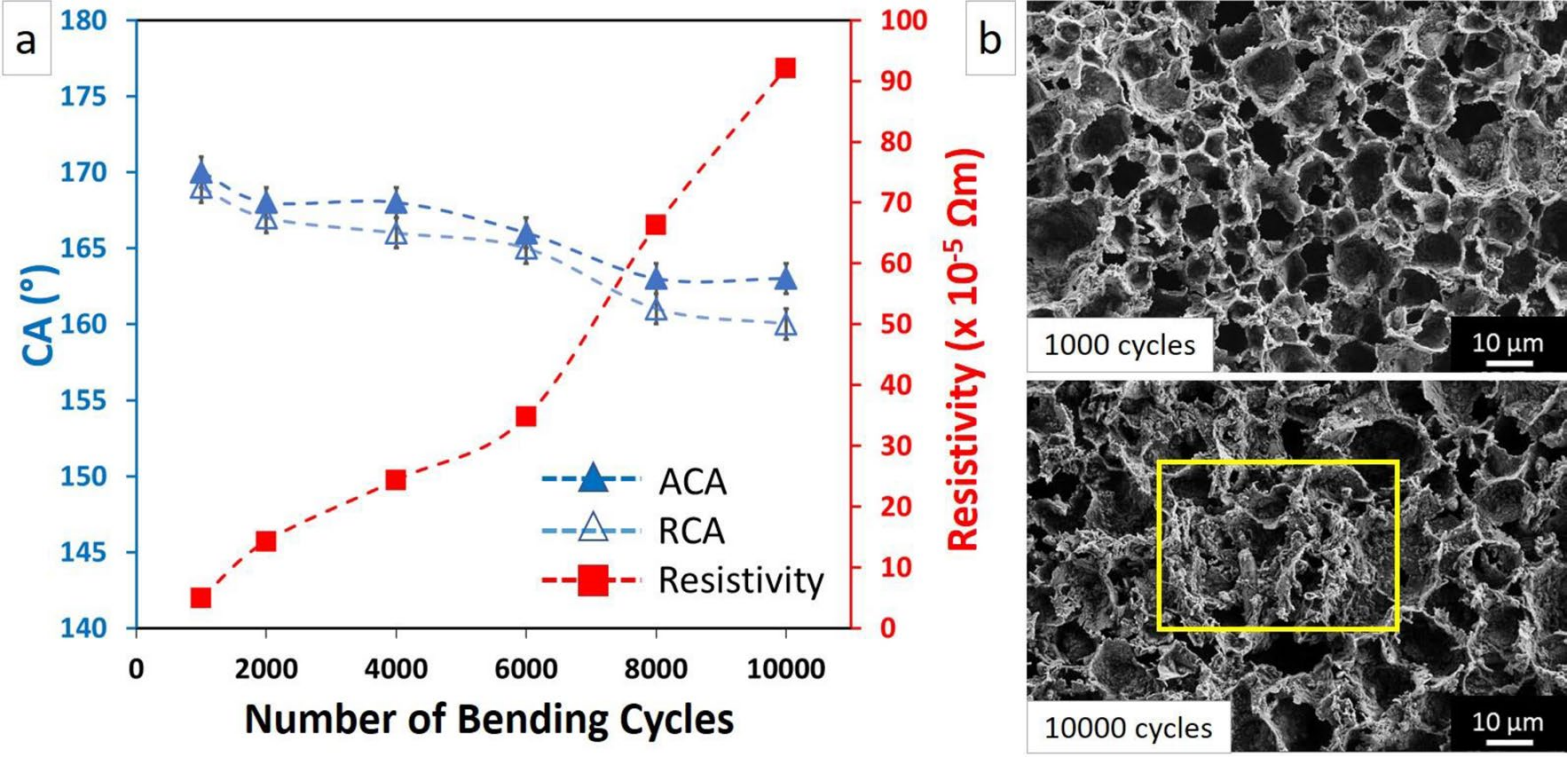
(**a**) The advancing, receding contact angles and resistivity after 10,000 bending cycles. (**b**) The SEM images of samples after 1000 and 10,000 cycles of bending.

Figure 6a shows the advancing, receding contact angles, and resistivity after cyclic bend test from 1000 to 10,000 cycles (Movie S3 in the supplementary material). The results show a small decrease in both advancing and receding contact angles after 10,000 bending cycles; the advancing contact angle decreased to 163 ± 1° and the receding contact angle was reduced to 160 ± 1°, so the superhydrophobic property of the material was preserved after cyclic bend test. The resistivity increased to 5 × 10^–5^ Ωm after 1000 bending cycles, and to 90 × 10^–5^ Ωm after 10,000 cycles due to deformation of hierarchical structures. The resistivity increase was more pronounced after 10,000 bending cycles compared to 10,000 stretching cycles. This is likely due to compressive failure that occurred in the bending test (Fig. 6b). Resistivity as a function of bending was not reported by Yao et al. and Asthana et al.^29,45^ As can be seen in Fig. 6b, the surface did not show any changes after 1000 cycles, while significant deformation in the surface structures can be observed after being subjected to 10 000 bending cycles.

## Conclusions

We introduce a simple and low-cost method to fabricate elastic, conductive, and robust superhydrophobic PDMS/Cu composite material. We studied the replication process of PDMS/Cu from structured aluminum and showed that two-step etching with native oxide removal and nanostructure formation in separate steps results in best topography.

Our process displays four major benefits: (i) it copies rigorously the nanostructures of aluminum; (ii) the deposition process is facile; (iii) we have a thick and durable conductive layer; (iv) overhang structures can be made. The first aspect is missing from simple polymer copying processes: they have difficulty reproducing the finer details of nanostructures. The second aspect is important for scaling to larger areas since electroless plating of copper is a very simple and cheap process that can be scaled up. The third aspect concerns both simplicity and durability of conductivity: thick copper film provides conductivity which cannot be achieved with e.g., silver nanoparticles or CNTs. The fourth aspect is important for oleophobicity: complete sacrifice of the aluminum substrate allows overhanging (retrograde) structures to be copied and released. Robust oleophobicity, however, remains to be demonstrated.

Superhydrophobic, conductive, elastic films can find applications in e.g., antibacterial surfaces. Superhydrophobicity in itself can reduce bacterial adhesion due to reduced contacts on nanostructured surface, and conductivity and elasticity can be used for electrical, electro-thermal or mechanical detachment of biofilms. Since PDMS self-adhesive and its thickness can be controlled, the material could function as conductive superhydrophobic tape.

It was found that the wetting properties of the resulting material can be optimized by choosing optimal etching processes and electroless plating parameters. These determine the surface topography as well as the thickness and structure of the copper layer and therefore control both the superhydrophobicity and the conductivity of the sample. It was also uncovered that the thickness of the copper layer leads to a trade-off between conductivity (the thicker, the better) and superhydrophobicity (which starts to decrease for thicker copper films beyond a certain point).

The superhydrophobicity and the conductivity of the PDMS/Cu material were resistant toward stretching, abrasion, and bending. We showed that the material remained superhydrophobic and conductive, even after subjecting to 10,000 cycles of stretching at 50% of tensile strain and 1000 cycles of abrasion. The reason for the durable superhydrophobicity is likely the millimeter-thick PDMS backing layer. Diffusion of PDMS oligomers to copper surface provides self-healing which is expressed as persistent superhydrophobicity. While there was a gradual increase in resistivity, it always remained below 90 × 10^–5^ Ωm, even after 3000 abrasion cycles or 10,000 stretching or bending cycles.

This replication method can be extended to other polymers (e.g., polyurethanes) and metals (e.g., nickel or silver) to fabricate novel superhydrophobic metal/polymer composite materials with different combinations of mechanical–electrical-fluidic-antibacterial properties.

## Methods

### Surface morphology and elemental analysis

Scanning electron microscopy (SEM, EBL Zeiss Supra 40) and energy-dispersive X-ray spectroscopy (EDX, Hitachi TM-4000Plus) were used to study on morphology and elemental composition of the resulting material. The thickness of plated copper and PDMS backing layer were measured from SEM cross-sections.

### Resistance measurements

To determine the sheet resistance, the four-point contact measurement was done with the help of an in-house built jig in which the electrical contacts were made with four brass screws. To achieve suitable contact, weight was added onto the jig until the resistance value stabilized, at which point we interpret that the contact resistance was minimized. Then, the resistance was measured by a digital multimeter (U3402A, Keysight). The resistance values were calculated as averages of three measurements.

### Contact and sliding angle measurement

The advancing and receding contact angles were measured through the sessile drop technique (THETA, Biolin Scientific). Advancing contact angles were measured from 2 to 5 μL droplet size and receding angels from 5 to 0 μL with a droplet pumping rate of 0.1 μL s^−1^. Sliding angles were measured with an in-house built goniometer with 7 μL water droplet. Both CAs and SAs were calculated as averages of three measurements.

### Mechanical durability tests

An in-house built test bench was used for stretching, abrasion, and bending tests. For a static stretch test, samples were stretched and fixed at 25% and 50% of tensile strain. To study the effect of stretching cycles, a cyclic stretch test was applied with two series, one at 25% and the other at 50% of tensile strain. The abrasion tests were conducted with silicon carbide paper (P 1200, Struers Co.), and the samples were abraded for a different number of cycles. The material also was subjected to cyclic bending tests.

## Supplementary Information

Supplementary Legends.

Supplementary Video 1.

Supplementary Video 2.

Supplementary Video 3.

Supplementary Video 4.

Supplementary Video 5.
