# Supplementary material for: Fabrication of elastic, conductive, wear-resistant superhydrophobic composite material
Source: Sci Rep. 2021 Jun 16;11:12646. doi: 10.1038/s41598-021-92231-x (PMC8209028; doi:10.1038/s41598-021-92231-x)
Supplement: Supplementary file 1 — Supplementary Legends. [file 41598_2021_92231_MOESM1_ESM.docx]

Movie S1: Stretch test

Movie S2: Abrasion test

Movie S3: Bending test

Movie S4: Knife test

Movie S5: Manual twisting and turning
